# Neoantigen immunotherapy: a novel treatment for bladder cancer

**DOI:** 10.37349/etat.2025.1002288

**Published:** 2025-01-26

**Authors:** Ruiyang Lv, Zhenzhu Liu, Maoxin Lv, Yuze Song, Junlin Wang, Huizhi Mu, Yu Zhang, Xuejian Wang

**Affiliations:** Université Paris-Saclay, France; ^1^Department of Urology, First Affiliated Hospital of Dalian Medical University, Dalian, Liaoning Province 116021, China; ^2^Department of Cardiovascular, Second Affiliated Hospital of Dalian Medical University, Dalian, Liaoning Province 116027, China; ^3^Department of Urology, First Affiliated Hospital of Kunming Medical University, Kunming, Yunnan Province 650032, China; ^4^Department of Nursing, First Affiliated Hospital of Dalian Medical University, Dalian, Liaoning Province 116021, China; ^5^Department of Anesthesiology, First Affiliated Hospital of Dalian Medical University, Dalian, Liaoning Province 116021, China

**Keywords:** Bladder cancer, immunotherapy, neoantigens

## Abstract

Bladder cancer is currently the most common malignant tumor of the urinary system. The traditional treatment methods for bladder cancer are mainly surgery, chemotherapy, radiotherapy, and targeted therapy; however, these treatment methods do not improve the clinical prognosis of patients with advanced or metastatic bladder cancer. Consequently, there is an urgent need to develop new treatment methods to improve the survival rate and quality-of-life of patients with bladder cancer. Over recent years, the rapid development of tumor immunotherapy has become a significant alternative to traditional treatment, and provides new hope to patients. This review aims to introduce neoantigens and their possible role in the treatment of bladder cancer, and to explore the current limitations of neoantigens for the treatment of bladder cancer.

## Introduction

Bladder cancer is one of the top ten most common cancers in the world today and is also the most common malignant tumor of the urinary system. This disease is three-fold more common in males than females [1, 2]. There are many causes of bladder cancer such as smoking, long-term exposure to benzidine, polyaromatic hydrocarbons, diesel exhaust gas, industrial chemical products, bladder stones, long-term repeated schistosomiasis infection, chronic bladder infection caused by long-term indwelling urinary catheters, and long-term irritation caused by foreign bodies. Of these, cigarette smoking is the most important risk factor for increasing the incidence of bladder cancer, and their replacement with electronic cigarettes has not reduced the incidence of this disease. In addition, environmental factors, such as advanced age, drinking water with a high arsenic content, ionizing radiation, and genetic factors, have all been shown to increase the incidence of bladder cancer [3–5].

Research has shown that 30% of bladder cancer cases invade the muscle layer at initial diagnosis with high metastasis recurrence; the other 70% of NMIBC (non muscle-invasive bladder cancer) patients also have a tendency to recurrence [6]. At present, different treatment options can be selected depending on whether the tumor has invaded the muscular layer. For intermediate-risk and more severe NMIBC patients, adjuvant therapy is typically supplemented with intravesical chemotherapy drugs or BCG (Bacillus Calmette-Guérin), to reduce the likelihood of tumor recurrence after surgery. This approach may also lower the risk of progression to MIBC (muscle-invasive bladder cancer) [7–9]. However, the current treatment outcomes for NMIBC remain suboptimal, with high recurrence rates still posing a significant challenge, indicating a need for new therapeutic strategies to improve the situation [10, 11]. Furthermore, when tumors progress to MIBC (T2–T4), radical cystectomy with lymph node dissection is considered a good option, with at least four cycles of cisplatin chemotherapy recommended prior to cystectomy to increase survival rates and reduce the risk of local recurrence and distant metastasis [12, 13]. In patients with MIBC who are not eligible for cisplatin, early-stage radical cystectomy remains the preferred treatment modality [14]. For patients whose physical conditions preclude surgery or who have metastases, treatment is mostly focused on palliative care or polyplatin. Polyplatin is the product of linking platinum-based drugs to nanomaterials and fluorescence, which can enhance the therapeutic effect, reduce side effects, and also enable diagnostic functions. The advent of immunotherapy has provided new hope for the treatment of bladder cancer. Currently, there are several promising clinical outcomes, particularly with immune checkpoint inhibitors (ICIs), which continue to show efficacy for the treatment of advanced bladder cancer [15, 16]. Patients who cannot tolerate chemotherapy or who relapse after chemotherapy should be treated with ICIs [17]. However, immune-related adverse events (irAEs) remain a significant challenge, and there is considerable variability in treatment response among different patients [18]. Therefore, there is an urgent need to develop new therapies to reduce the recurrence rate of bladder cancer and improve patient survival.

The immunological “foreignness” of cancer has led to the development of new immunotherapies. Mutations in the DNA sequence of cancer cells can produce altered or entirely new protein products, which the immune system can recognize as “non-self”. These altered or novel proteins are antigenic to the host and are therefore referred to as neoantigens. The identification of cancer-related neoantigens has sparked significant interest in developing therapeutic vaccines that specifically target tumors while sparing healthy tissues. The responsiveness to ICI drugs is thought to be associated with the tumor mutational burden (TMB) [19, 20]. As a result, neoantigens have garnered attention in recent years as potential candidates for therapeutic vaccines designed to enhance existing endogenous T-cell responses or elicit de novo T-cell responses against these neoantigens, thereby boosting anti-cancer immunity to clinically relevant levels and improving or extending the efficacy of immune checkpoint blockade (ICB). However, the potential of neoantigen-based therapies in bladder cancer immunotherapy has not been thoroughly explored. This review aims to summarize recent advances in neoantigen-related research within bladder cancer immunotherapy, highlighting their advantages and challenges.

## Immunotherapy and bladder cancer

The body’s immune system features two components: innate immunity and adaptive immunity. Innate immunity can identify and eliminate foreign pathogens without prior exposure to pathogens. Natural killer cells are important cells in this form of immunity; these cells can be directly activated by their lack of major histocompatibility and then proceed to kill target cells. Adaptive immunity is driven by T cells and B cells [21].

The body’s immune system has a strong ability to clear tumor cells, although tumor cells can disguise themselves by changing their phenotype to avoid the pursuit of immune cells or produce a tumor microenvironment to suppress immune cells [22]. Therefore, the key to treating bladder cancer is to give full consideration to the full capability of the human immune system.

ICIs have developed rapidly over recent years and have become the most effective form of cancer immunotherapy. ICIs are monoclonal antibodies that target inhibitory immune checkpoints, which are exploited by cancer cells to escape the immune system. Approved ICIs include inhibitors of programmed cell death 1 (PD-1), programmed cell death ligand 1 (PD-L1), cytotoxic T lymphocyte antigen 4 (CTLA-4), and lymphocyte activation gene 3 [23, 24]. Current Food and Drug Administration (FDA)-approved immunodrugs for metastatic urothelial bladder cancer include PD-L1 inhibitors such as atezolizumab, durvalumab and avelumab, and PD-1 inhibitors, including pembrolizumab and nivolumab. These drugs are mainly used as second-line therapy for patients with locally advanced or metastatic urothelial carcinoma who have progressed within 12 months of treatment while receiving platinum-based combination chemotherapy. Atezolizumab and pembrolizumab may also be used as first-line therapeutics in patients with advanced urothelial carcinoma who cannot tolerate platinum-based chemotherapy and are PD-L1-positive. ICI monotherapy, or in combination with other anti-cancer treatments, can improve anti-tumor efficacy for many types of cancer; however, the therapeutic effect on bladder cancer remains disappointing [25–28]. In addition to the recent rise of ICIs, there is also a long history of treating bladder cancer with immunotherapy methods, such as BCG.

BCG is a live and attenuated form of *Mycobacterium tuberculosis* of bovine origin and was first cultured by Calmette, a French scientist, in 1908. BCG has been used for a long period of time as a vaccine with which to prevent tuberculosis. The anti-tumor potential of BCG was first reported in 1929, when Pearl performed autopsies and detected a lower incidence of tumors in patients with tuberculosis. In 1976, Morales was the first to successfully use BCG bladder instillation to treat bladder cancer; this protocol significantly reduced the rate of postoperative recurrence in patients. In 1980, Lamm performed a prospective study and confirmed the therapeutic role of BCG in bladder cancer. In 1990, BCG was approved by the US FDA for bladder instillation, and became the world’s first approved tumor treatment that was immunoresponse-based. Subsequently, multi-center clinical controlled studies have proven that BCG is the preferred drug for postoperative perfusion therapy for the treatment of medium- and high-risk NMIBC. The precise mechanism of action underlying the effect of BCG in the treatment of bladder cancer remains unknown, although it is generally believed that BCG acts as an immunomodulator and causes the body to produce a specific immune response to destroy tumor cells. BCG bladder infusion therapy is currently the only therapy that can reduce the recurrence and progression of bladder tumors after surgery; other chemoperfusion drugs (such as epirubicin, pyrrubicin, and mitomycin) can reduce the recurrence rate of tumors but cannot prevent tumor progression. As the most mature immunotherapy for bladder cancer, BCG still exposes many problems, including intolerance, resistance, recurrence, and other serious adverse reactions [29–33].

Novel antigen-based tumor vaccines appear to be more widely applicable when compared to BCG; this is because bladder cancer has two main characteristics. First, bladder cancer is associated with a high somatic mutation rate. Previous studies have shown that bladder cancer has an average somatic mutation rate of 7.7 per megabase, second only to melanoma and non-small cell lung cancer [34]. This high mutation rate can produce many neoantigens for the immune system to recognize and stimulate the immune response. Secondly, this condition can easily relapse. Many researchers have observed the recurrence and progression of NMIBC, with a 5-year probability of recurrence and progression of 78% and 45%, respectively, following the transurethral resection of bladder tumors (TURUBTs) [4]. Thus, postoperative treatment, involving the infusion of drugs, is usually required to reduce the recurrence rate. However, 20% to 30% of patients will still experience recurrence, and approximately 20% of patients will develop MIBC after recurrence. In patients with MIBC, 50% of surgical patients will still experience disease recurrence within two years of diagnosis [35]. However, precisely because bladder cancer recurrence has a long time window, within this time period, we can screen out tumor antigens and make vaccines from the tumor samples obtained from the first TURUBT surgery. The selected antigen is critical and is the key to the success of a vaccine.

## Neoantigens

Neoantigens are a class of tumor-specific antigens (TSAs) that are expressed only in tumor tissues and not in normal tissues. Compared with tumor-associated antigens (TAAs) expressed in both tumor and normal tissues, neoantigens exhibit stronger immunogenicity and higher affinity toward MHC (major histocompatibility complex), and are not affected by central immunological tolerance. Neoantigens are highly individual-specific and usually do not involve known oncogenes. The larger the difference between mutation sequence and original coding sequence, the more obvious the “non-self” feature of the abnormal protein and stronger the immunogenicity. Whether mutations can form tumor neoantigens depends on several factors: 1) whether the mutated se-quence can be translated into protein; 2) whether the mutated protein can be processed into peptides and pre-sented; 3) affinity between the mutated peptide and MHC molecules of the patients; 4) affinity of mutant peptide-MHC complex with T cell receptor (TCR). In addition, neoantigens can activate CD4^+^ and CD8^+^ T cells to generate an immune response and have the potential to become new targets of tumor immunotherapy [36].

### Source of neoantigens

There are four main sources of neoantigens. First, the insertion and deletion of nucleotides (INDELs) may lead to the production of new peptides; these may also induce immunity [37]. Secondly, intra-chromosomal and inter-chromosomal rearrangement may cause two unrelated genes to fuse to generate a fusion gene, although the vast majority of fusion genes are individual [38]. Third, alternative splicing produces diversity and lineage-specificity by expressing multiple RNA and protein isoforms from one gene, thus causing dysregulation in tumor cells and the generation of new epidermis-specific sequences [39, 40]. Fourth, non-coding genomic regions and RNA editing may also produce new peptides. Based on their potential functional impact, neoantigens can also be divided into protective neoantigens that are cross-recognized by pre-formed memory T cells induced by heterologous immunity, restricted neoantigens that are immunogenic in immunotherapy hosts and induce PD-1^+^ memory T cells, and neglected neoantigens that do not induce associated immune responses in tumor-containing hosts but can effectively promote tumor immunity once memory T cells are induced by vaccination [41].

### Recognition of neoantigens

To stimulate an anti-tumor immune response, neoantigens need to be processed and presented by MHC class I and II molecules, recognize TCRs, and then stimulate T cell activation and infiltration [42]. The MHC-I molecule is expressed by almost all cell types and helps to eliminate infected or transformed cells such as bacteria, viruses, or mutant peptides. The MHC-II molecule is predominantly presented in antigen-presenting cells (APCs), such as dendritic cells (DCs) and macrophages. By assisting CD4^+^ T lymphocytes, the MHC-II molecule supports the development of cytotoxicity and humoral immune responses. In response to malignant transformation, cells may also produce proteins with tumor-specific post-translational modifications, such as phosphorylation; some of these proteins can be loaded onto MHC-I molecules for presentation to CD8^+^ T cells [43]. The enormous peptide diversity exhibited by MHC-I and MHC-II molecules on all somatic cells complements the significant TCR diversity generated by genetic recombination events in CD8^+^ and CD4^+^ T lymphocytes. Together, these events confer the immune system’s ability to monitor immunity and respond to highly diverse pathogens and tumor neoantigens [44].

### Neoantigen-driven immune effector mechanisms

**Figure 1:**
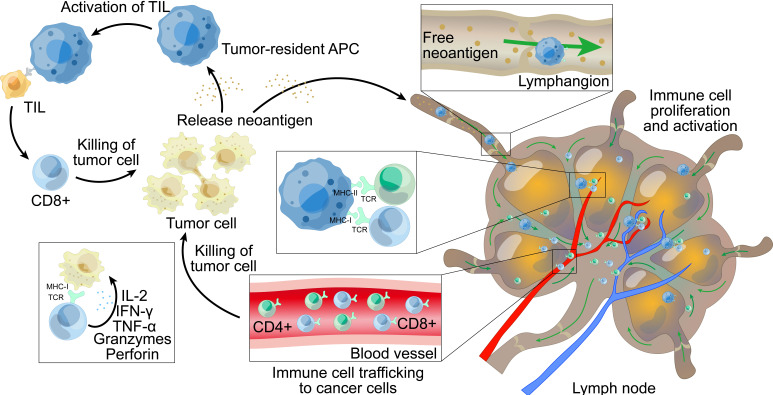
**The main mechanism of immune response induced by neoantigens**. (1) Inside the tumor, neoantigens released by tumor cells are presented to TILs (tumors infiltrate lymphocytes) by tumor-resident APCs. Some TILs can specifically recognize neoantigens and proliferate and activate after recognition, thereby attacking and killing tumor cells, and releasing more neoantigens after the death of tumor cells. (2) Some neoantigens are released from the tumor tissue and are taken up by APCs in the lymphatic vessels, which present the neoantigens to T cells in the lymph nodes, triggering T cells to proliferate and activate. The activated T cells are then transported to the tumor tissue through the blood vessels and kill the tumor cells; the death of tumor cells causes the release of more neoantigens. MHC: major histocompatibility complex; TCR: T cell receptor; IFN-γ: interferon-γ; APC: antigen-presenting cell

CD8^+^ and CD4^+^ T cells can differentiate into tumor-infiltrating PD-1^+^ effectors and memory T cells after activation. In the tumor microenvironment, activated neoantigen-specific T cells fulfill their effector function by recognizing their neoantigens on intra-tumor APCs and tumor cells; this reaction contributes to tumor control [45, 46]. CD4^+^ and CD8^+^ T cells induce the upregulation of MHC-I and MHC-II expression on tumor cells and APCs following the recognition of homologous antigens; this can help to enhance our understanding of neoantigen recognition [47, 48]. The death of tumor cells, along with the release of tumor antigens, then lead to the further spread of antigens which stimulate the release of further CD4^+^ and CD8^+^ T cells. The levels of MHC-I presented neoantigens correlate with the expression of neoantigens; higher levels of presentation may trigger an immune response that may lead to the downregulation of the mutated allele of the neoantigen or removal of the tumor [49]. The specific process of neoantigens causing an immune response is shown in Figure 1.

### Current clinical applications of neoantigens in cancer immunotherapy

At present, our understanding of neoantigens is developing rapidly; furthermore, neoantigens have gradually begun to be used effectively for the treatment of various tumors. Neoantigen immunotherapy has been shown to control the growth of gastric tumors. Furthermore, the recognition of neoantigens can monitor the dynamics of tumor immune response and help to evaluate the impact of combination immunotherapy on tumor-specific immune response. These mechanisms will help us to develop novel combination immunotherapies for the treatment of gastric cancer [50]. Because neoantigens can be recognized by tumor-infiltrating cytotoxic CD8^+^ T cells [51], the presentation and burden of neoantigens are both positively correlated with the prognosis of patients with various cancers [52–54]. The extensive clonal mutation that exists in smoking-associated non-small cell lung cancer may also make tumors susceptible to vaccination against polyclonal neoantigens or T-cell therapy [55]. In preclinical models, a low mutational load has been shown to lead to a lack of immune editing in mouse KPC pancreatic cancer models, while the introduction of neoantigens can lead to tumor reduction and eradication [56, 57]. In addition, researchers have developed a personalized neoantigen vaccine by identifying neoantigen designs, and by isolating immune cells from patients who specifically recognize neoantigens. These cells are then modified in vitro and then reinjected into patients to fight tumors. This strategy can help us to develop personalized vaccines or immune cells that specifically identify neoantigens for the treatment of malignant melanoma, metastatic breast cancer, and glioblastoma, thereby reducing the recurrence rate of tumors and helping us to achieve the clinical treatment of advanced tumors [58–60]. With regards to the treatment of Lynch syndrome (hereditary non-polyposis colorectal cancer), studies have shown that mice injected with neoantigen vaccines experience a longer survival time than mice that did not receive an injection, thus providing a new direction for the treatment of hereditary diseases. In addition to these tumor types, personalized vaccines have also achieved promising results in the treatment of ovarian cancer, soft tissue sarcoma, head and neck squamous cell carcinoma, and other forms of tumors [61, 62].

## Neoantigens and bladder cancer

### Neoantigen-based therapeutic strategies

Over recent years, neoantigen-based immunotherapy has begun to show efficacy for the treatment of some tumors. Some neoantigen-based immunotherapies have also been used to treat bladder cancer.

Adoptive cell therapy (ACT) refers to the transfusion of autologous or alloimmune effector cells (that are activated and expanded in vitro) into patients, including tumor-infiltrating lymphocytes (TILs) therapy, TCR-T therapy and CAR-T therapy. TCR-T cell therapy was first used in clinical practice in 2006. In 2014, the targets of TCR-T cell therapy were extended to individualized neoantigens. At present, individualized neoantigen TCR-T therapy provides a relatively mature for of screening and is associated with an established preparation protocol; furthermore, some progress has been made in the treatment of solid tumors. Five TCR-T clinical trials are ongoing, although none of these trails feature patients with bladder cancer [63]. As solid tumors, bladder tumors share some of the same characteristics as other solid tumors, and patients with bladder cancer may soon participate in clinical trials as subjects. CAR-T is associated with some limitations with regards to the treatment of solid tumors, and no significant progress has been made in terms of CAR-T [64]. Previously, researchers isolated neoantigen-reactive TIL from melanoma and gastrointestinal cancer; this led to sustained tumor regression when amplified in vitro and transferred back to the patients, thus confirming that TIL could be successfully produced by bladder tumors. The researchers attempted to identify the mutated form of C-terminal-binding protein 1 (CTBP1Q277R) from CD4^+^ TIL cells harvested from patients with bladder cancer in an MHC class II restricted way. The researchers identified a CTBP1 mutation at amino acid 277; this has yet to be reported in the Catalogue of Somatic Mutations in Cancer (COSMIC) database and therefore appears to be unique to this patient. This suggests that TILs can be produced by primary bladder tumors, and that neoantigen-reactive TIL can also be isolated from bladder tumor specimens, thus providing preliminary evidence for the use of adaptive metastatic neoantigen-reactive T cells for the treatment of patients with bladder cancer [41].

Antibody-drug conjugate (ADC) therapy and bispecific antibody therapy have received significant levels of research attention over the past year; in addition, several studies have linked neoantigens to ADC therapies. An anti-MUC1 ADC was previously synthesized by conjugating GSTA neoantigen-specific 16A with monomethyl auristatin E (MMAE); this drug exhibited potent antitumoral efficacy with an IC_50_ ranging from 0.2 nM to 49.4 nM towards various types of cancer cells [65]. This data suggested that neoantigen-specific ADC therapy could be used to treat cases of bladder cancer in the future.

### Neoantigen vaccine and bladder cancer

Neoantigens can be divided into two categories: shared neoantigens and personalized neoantigens [66, 67]. Shared neoantigens are mutated antigens that are common in patients with different types of cancer but are not present in the normal genome. Highly immunogenic shared neoantigens have the potential to be used to synthesize broad-spectrum cancer vaccines for patients with the same mutated genes [68, 69]. Individualized neoantigens refer to mutated antigens that are unique to patients with different types of cancers. Personalized neoantigen vaccines can only specifically target the corresponding individual patient, and can be used alone or in combination with other therapies to increase a patient’s immunity. Many different types of cancer vaccines are available, including peptide vaccines, DNA vaccines, mRNA vaccines, and DC vaccines.

In recent years, there has been more exposure of neoantigen vaccines to urological tumors. A research team has identified TOP2A, NCF4, FMNL1 and DOK3 as potentially effective neoantigens for the development of *KIRC* mRNA vaccines, and divided renal cell carcinoma into two immune subtypes, RIS1 and RIS2. Studies have shown that patients with RIS2 tumors may be able to achieve better treatment efficacy when administered mRNA vaccines [70]. The same research team identified KLHL 17, CPT 1B, IQGAP 3, LIME 1, YJEFN 3, KIAA 1529, MSH 5 and CELSR 3 as potential antigens for *PRAD* mRNA vaccine development, and divided prostate cancer into three immune subtypes (PIS1, PIS2 and PIS3). Tumor patients with the PIS2 and PIS3 types may be more suitable for treatment with mRNA vaccines [71]. At present, the use of neoantigens for the treatment of urinary tumors is relatively limited. Trials of a personalized vaccine (NeoVax) for the treatment of kidney cancer are actively underway. Furthermore, a neoantigen DNA vaccine combined with nivolumab/ipilimumab and Prostec for the treatment of metastatic hormone-sensitive prostate cancer has been trialed but failed to yield positive results. The use of neoantigens for the treatment of bladder cancer appears to have progressed further than kidney and prostate cancer. There are some ongoing and complete clinical trials related to personalized neoantigen vaccines for bladder cancer completed.

**Figure 2:**
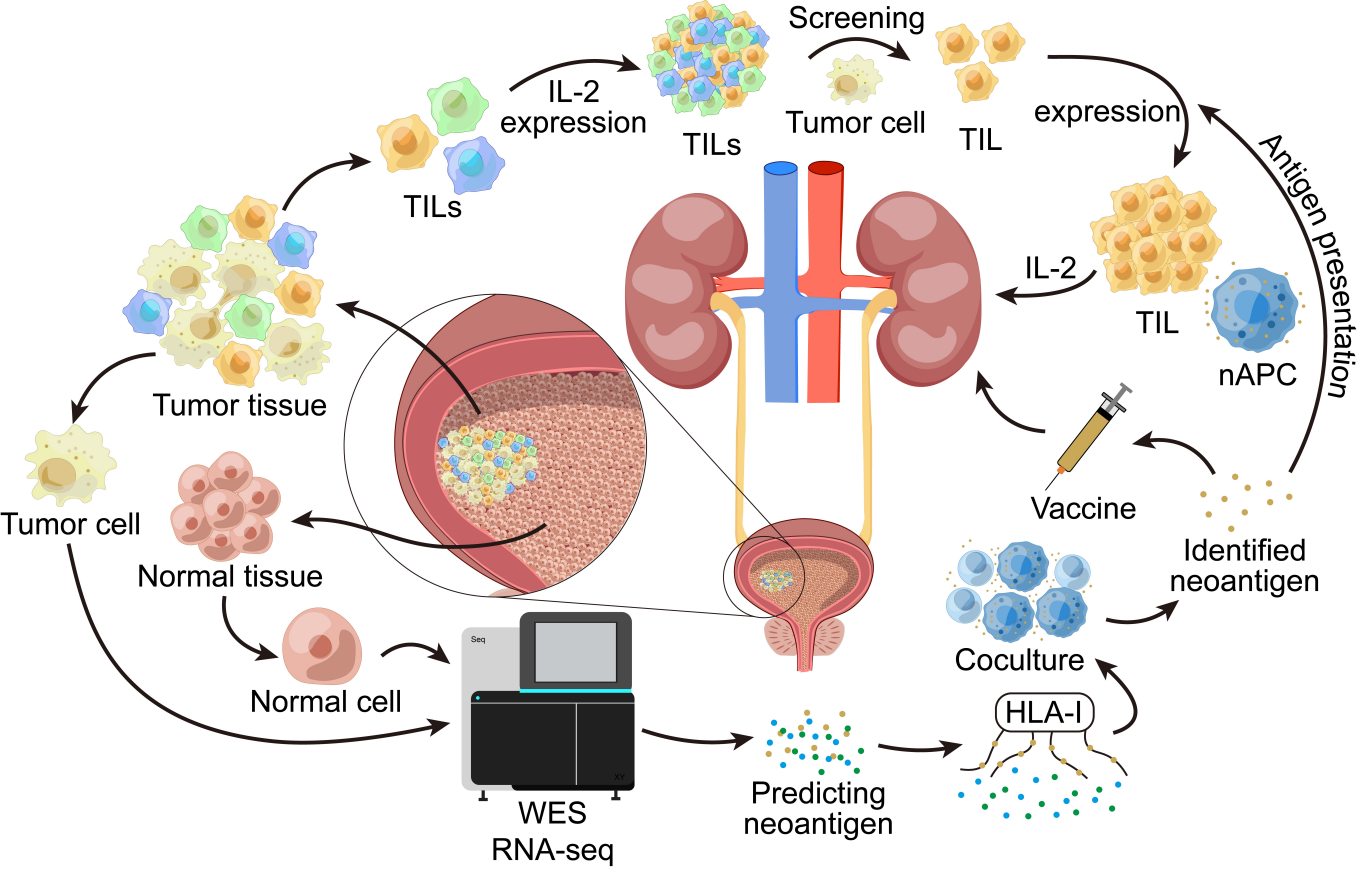
**WES and RNAseq are performed on tumor cells and normal cells in the bladder**. This allows the prediction of possible neoantigens. Then we measure the affinity of the predicted neoantigens with HLA-I, select the neoantigens with the highest affinity to HLA-I and then co-culture APCs loaded with this neoantigen with the patient’s T cells in vitro to verify whether the neoantigen can effectively activate the T cell immune response. The validated neoantigens can be used to make vaccines and treat patients. TILs: tumor-infiltrating lymphocytes; nAPC: antigen-presenting cell carrying neoantigens; HLA-I: human leukocyte antigen I; WES: whole exome sequencing; RNAseq: RNA sequencing; APCs: antigen-presenting cells

A previous study reported a first Phase 1b clinical trial of an individualized neoantigen-based vaccine (NEO-PV-01) combined with a PD-1 blocker for the treatment of patients with bladder cancer. A total of 15 bladder cancer patients were vaccinated, and 11 of them completed the full course of treatment. The NEO-PV-01 vaccine is personalized to each patient’s unique tumor gene mutation load. WES (whole exome sequencing) and RNAseq (RNA sequencing) are performed on the patient’s tumor cells and normal cells first. High-quality neoepitopes encoded by somatic mutations were then selected using bioinformatics algorithms. Finally, the affinity of the predicted neoantigen to HLA-I (human leukocyte antigen I) was measured, and the neoantigen with the highest affinity for HLA-I was selected, and the neoantigen vaccine was produced (Figure 2). While the vaccine was being produced, the patient was treated with nivolumab for 12 weeks. From week 12 onwards, patients received five vaccinations and three immune boosts, which make up a full NEO-PV-01 course. Nivolumab was continued during both the vaccine and post-vaccine time periods.

The experimental principle is that the infusion of NEO-PV-01 activates T cells in the human body to produce an anti-tumor immune response, thereby killing tumor cells. In experiments, the researchers observed that post-vaccine peripheral CD4^+^ T cells upregulated CD107a in response to stimulation with many of the vaccine neoantigen peptides, indicating their cytolytic capacity. Moreover, the vaccine-generated T cells exhibit a memory phenotype, have cytotoxic potential, are mutation-specific and persistent, and can migrate to tumors. In addition, researchers detected epitope spread to neoantigens not included in the vaccine after vaccination. This suggests that the neoantigen-induced T cells produced by the NEO-PV-01 vaccine are not only transported to the tumor; they may also kill tumor cells, thereby releasing additional neoantigens that become targets for further T cells. The spread of epitopes may be a self-amplifying process in which dead tumor cells can trigger an additional neoantigen-specific immune response during treatment, and its production may help control tumor cells that do not express backbone neoantigens, thus possibly a mechanism to control tumor heterogeneity. Experimental results showed that the median PFS of vaccinated bladder cancer patients was 5.8 months (2.8, 12.7), the median OS was 20.7 months (4.8, NE), and the one-year survival rate was 67% (38–85%). The combination of NEO-PV-01 with a PD-1 blocker significantly prolonged survival when compared with PD-1 blocker monotherapy. Clinical data suggest that it is safe and feasible for bladder cancer patients to receive anti-PD-1 therapy and neoantigen vaccination at the same time. This study laid the groundwork for the use of a personalized vaccine for the treatment of bladder cancer, although the study has limitations. Since the treatment was a combination of the neoantigen vaccine and anti-PD-1, there was a lack of controlled trials for the use of anti-PD-1 alone, and supplemental controlled trials are necessary to demonstrate the specific effects of personalized neoantigen vaccines [72].

Researchers previously developed a personalized vaccine that was called PGV-001; this is an adjuvant treatment vaccine that is used following standard treatment. The main function of this vaccine is to remove remaining cancer cells from the body and prolong the survival of patients. A total of 15 patients were enrolled, and all patients with solid tumors underwent radical surgery, including one patient with bladder cancer. An average of 67.1 neoantigens per patient (range 8–193) were detected by sequencing and bioinformatics algorithms. Thirteen of the 15 patients received PGV-001 the other two patients were unable to receive treatment because of the lack of neoantigens to produce the vaccine. Eleven patients received 10 custom-designed vaccinations over a six-month period, among those treated with the vaccine. Furthermore, the patients tolerated the drug well, experienced no serious side effects, and achieved good treatment outcomes. After six injections, there was no obvious immune response, and after 10 injections, a strong anti-cancer response of the immune system was observed. Following vaccination, the researchers followed the patients for approximately 880 days; during this time period, there were four patients with no signs of disease and no follow-up treatment, including one with bladder cancer. These results indicated that personalized neoantigen vaccines may be an effective strategy for treating bladder cancer [73]. Further clinical trials involving neoantigen vaccines for the treatment of bladder cancer are currently under way (Table 1).

**Table 1:** Clinical trials involving neoantigen vaccines for the treatment of bladder cancer

**Identifier**	**Number of subjects**	**First submitted date**	**State**
NCT03715985	12	October 17, 2018	-
NCT03359239	10	November 27, 2017	Completed
NCT05269381	-	February 25, 2022	Recruiting
NCT03794128	93	April 8, 2019	Completed
NCT03639714	29	August 6, 2018	Completed
NCT05235607	-	August 24, 2019	Not yet recruiting
NCT03633110	24	August 6, 2018	Completed

### Challenges and future prospects of neoantigen therapy

Owing to the discovery of ICI therapy, research is increasingly focusing on the role of the immune system in cancer treatment [74]. Problems have also arisen in the context of neoantigen vaccine combination immunotherapy. For example, the sequences of different preparations in the treatment process need to be investigated further to improve the efficacy of clinical treatment. In addition, whether ICI treatment alone and neoantigen vaccine treatment alone can improve the efficacy of clinical treatment also needs to be proven by randomized studies. Finally, neoantigen-specific T cell response assays need to be performed in patients who do not respond to combination therapy to modulate T cell function and thereby improve treatment efficacy [75]. The acquisition of neoantigens is also a problem. Acquiring neoantigens efficiency is necessary because we have to acquire neoantigens and make vaccines in a limited time, which depends on advances in sequencing technology. At the same time, the quality of the neoantigens directly determines the efficacy of cancer vaccines, which suggests that we should better use bioinformatics algorithms better. Some patients may lack enough neoantigens to make the vaccine. The randomness and significant heterogeneity of tumor neoantigens may significantly lengthen the preparation process and increase the cost of immunotherapy products such as cancer vaccines, further limiting their clinical application. These are the problems that we need to face if we are to apply neoantigen treatments on a wider basis.

The success of cancer immunotherapy highlights the powerful ability of local adaptive immune responses to eradicate cancer cells via neoantigens produced by somatic cell alterations. However, the screening for targets that are recognized by specific T cells in neoantigens produced by the large number of mutations associated with bladder cancer is a huge undertaking. Furthermore, the heterogeneity of bladder tumors may be more pronounced than metastasis, thus preventing the recognition of TILs, recognizing mutations in specific parts of the tumor, and reducing the ability to identify neoantigen-response TILs in bladder cancer. Furthermore, the high loss rates of MHC class I molecular modified in primary bladder tumors limits the presentation of bladder tumor neoantigens [76, 77].

Research into neoantigen immunotherapies is currently underway around the world. The rapid development of high-throughput sequencing technology, including whole-genome sequencing and the whole-exon sequencing, which are now less expensive and more convenient than they have been in the past, has led to explosion of sequencing data and identification of thousands of tumor-associated genes. Synthetic peptide-based neoantigen vaccines have been used in multiple clinical trials and have demonstrated good safety and efficacy. Some early trials based on public neoantigen cancer vaccines have also reported good results. A major advantage of sharing neoantigens is that they can be rapidly applied to cancer patients, especially those with advanced cancer and those with narrow treatment windows. In addition, cancer vaccines with public neoantigens will reduce the cost of treatment. Neoantigen immunotherapy has shown promising potential to be one of the most effective strategies for treating cancer [78].

## Abbreviations

ADC: antibody-drug conjugate
APCs: antigen-presenting cells
BCG: Bacillus Calmette-Guérin
HLA-I: human leukocyte antigen I
ICIs: immune checkpoint inhibitors
MHC: major histocompatibility complex
MIBC: muscle-invasive bladder cancer
NMIBC: non muscle-invasive bladder cancer
PD-1: programmed cell death 1
PD-L1: programmed cell death ligand 1
RNAseq: RNA sequencing
TCR: T cell receptor
TILs: tumor-infiltrating lymphocytes
WES: whole exome sequencing

## Declarations

### Copyright

© The Author(s) 2025.
